# Remarks on High Blood Pressure and Arteriosclerosis

**Published:** 1912-02

**Authors:** 


Remarks on High Blood Pressure and Arteriosclerosis.—J. M. Jackson of Boston (*Bost. Med. & Surg. Journ.*, November 2, 1911), calls attention to the importance of routine measurement of blood pressure in persons entering upon or early in middle age. In these cases increased pressure is often the only sign of a chronic poisoning arising from deficiency of elimination, either intestinal or urinary. The increase need not be great to injure any ordinary heart in time. The intestinal tract is frequently responsible for the toxins either through frank constipation or slow and deficient elimination. He cites the case of a woman who had been under treatment for "neurasthenia" for ten years. She had been subject to frequent attacks of severe headache and profuse nosebleeds. The blood pressure was 230 during an attack. Although there had been a daily bowel movement, investigation showed that the source of the trouble was in the intestinal tract. Under appropriate treatment, the pressure was reduced and perfect health resulted.

Overwork is another potent cause of high tension and its sequel arteriosclerosis. It is found particularly among brain workers who are kept at a high pitch. There is also undoubtedly a hereditary tendency to high arterial tension. The symptoms of the condition may simulate almost any disease, but those that are often attributed to neurasthenia are especially mentioned. The changes in the retinal vessels are frequently the first indication of the increased pressure, so that the ophthalmologist is often the discoverer of the condition.

